# The surrounding landscape influences the diversity of leaf-litter ants in riparian cloud forest remnants

**DOI:** 10.1371/journal.pone.0172464

**Published:** 2017-02-24

**Authors:** Miguel Á. García-Martínez, Jorge E. Valenzuela-González, Federico Escobar-Sarria, Fabiola López-Barrera, Gabriela Castaño-Meneses

**Affiliations:** 1 Instituto de Ecología, A.C., Xalapa, Veracruz, México; 2 Ecología de Artrópodos en Ambientes Extremos, Unidad Multidisciplinaria de Docencia e Investigación, Facultad de Ciencias, Universidad Nacional Autónoma de México, *Campus* Juriquilla, Querétaro, México; Arizona State University, UNITED STATES

## Abstract

Riparian vegetation is a distinctive and ecologically important element of landscapes worldwide. However, the relative influence of the surrounding landscape on the conservation of the biodiversity of riparian remnants in human-modified tropical landscapes is poorly understood. We studied the surrounding landscape to evaluate its influence on leaf-litter-ant alpha and beta diversity in riparian remnants in the tropical montane cloud forest region of central Veracruz, Mexico. Sampling was carried out in 12 sites with riparian vegetation during both rainy (2011) and dry (2012) seasons. Ten leaf-litter samples were collected along a 100-m transect per site and processed with Berlese-Tullgren funnels and Winkler sacks. Using remotely-sensed and ground-collected data, we characterized the landscape around each site according to nine land cover types and computed metrics of landscape composition and configuration. We collected a total of 8,684 ant individuals belonging to 53 species, 22 genera, 11 tribes, and 7 subfamilies. Species richness and the diversity of Shannon and Simpson increased significantly in remnants immersed in landscapes with a high percentage of riparian land cover and a low percentage of land covers with areas reforested with *Pinus*, cattle pastures, and human settlements and infrastructure. The composition of ant assemblages was a function of the percentage of riparian land cover in the landscape. This study found evidence that leaf-litter ants, a highly specialized guild of arthropods, are mainly impacted by landscape composition and the configuration of the focal remnant. Maintaining or improving the surrounding landscape quality of riparian vegetation remnants can stimulate the movement of biodiversity among forest and riparian remnants and foster the provision of ecosystem services by these ecosystems. Effective outcomes may be achieved by considering scientific knowledge during the early stages of riparian policy formulation, in addition to integrating riparian management strategies with broader environmental planning instruments.

## Introduction

Riparian remnants are commonly encountered as linear strips of vegetation alongside watercourses and are recognized as important elements of human-dominated landscapes worldwide [1]. These remnants may have distinct species compositions that differ from those of the surrounding habitats [2]. Relative to their extent, they may act as disproportionate reservoirs for local and regional biodiversity [3]. These strips of vegetation are also recognized as important ecological corridors and are used in conservation planning to promote functional landscape connectivity [4]. Moreover, these linear forest remnants provide ecological services (i.e., interception of sediment, litter input, nutrient absorption and regulation of rainwater infiltration) of great value to the functioning of the ecosystem [5].

The conservation value of riparian vegetation remnants in maintaining biodiversity has been widely studied in forest, agricultural, and urban landscapes [6–11]. Most of these studies have focused on comparing diversity and changes in species composition at the habitat scale by assessing different riparian characteristics, such as width [8], structural complexity of vegetation [9,10], or degree of disturbance [3]. Other studies have compared biodiversity associated with riparian remnants with that of adjacent non-riparian habitats [7,12,13].

Furthermore, riparian remnants have been highly modified due to human activities, and they are particularly vulnerable to changes in the surrounding landscape due to their linear configuration (i.e., a high edge to area ratio) [14–16]. Due to this landscape feature, the biodiversity of riparian remnants is expected to be more influenced by the type of matrix (i.e., surrounding non-habitat land uses) [6–8]. Some studies have demonstrated that land uses/covers (LUCs) such as secondary forests, tree crops, or cattle pastures with isolated trees may have disproportionate benefits for the diversity of ants, bats, birds, and frogs associated with riparian remnants in anthropogenic regions [7,12,17,18]. These non-habitat LUCs support riparian biodiversity, acting as complementary and/or supplementary habitats where species can forage and obtain additional resources [19].

However, the relative influence of landscape patterns on riparian biodiversity is poorly understood [1,2]. A few studies have suggested that species diversity and native species abundance are negatively affected when the composition (i.e., covered proportion and number of different land uses) and the configuration (i.e., spatial arrangement of land uses) of the surrounding landscape are human-modified or fragmented, respectively [14–16]. Other studies have indicated that although riparian remnants have been highlighted as biodiversity refuges, these areas may have a reduced capacity to maintain species diversity in highly transformed landscapes [3,12,15]. Therefore, landscape patterns could be an important aspect to consider in management and conservation planning in order to promote functional landscape connectivity [4,8,16].

Useful information for conservation planning has often been based on the diversity of insect groups like ants. In most terrestrial ecosystems, ants are ecologically important and, according to their biological attributes, are very useful for evaluating and monitoring biodiversity and changes in biodiversity [20,21]. In fact, ants represent an excellent model taxon because they respond rapidly to environmental change, represent a variety of trophic levels, are important ecosystem engineers and agents for plant seed dispersal, and have been used effectively as ecological indicators [22,23].

Leaf-litter ants represent more than 50% of the total ant community in forest ecosystems and have high densities and species richness in tropical forest regions [24–26]. They play a major ecological role in these ecosystems as predators, fungus-growers, scavengers, or parasites and are sensitive to environmental changes [27]. At a habitat scale, some studies, carried out in riparian zones, indicate that a higher species diversity is positively related with a high diversity and structural complexity of the vegetation or environment quality [7,9,13,23]. At a landscape scale, ants can use different land-uses to obtain supplementary and/or complementary resources that may compensate for limited resource availability in natural habitats [28,29]. In fact, leaf-litter ants are influenced by variation in the composition and configuration of their surrounding landscape [30–32]. For this guild of ants, the landscape composition may serve as an indicator of resource availability and environmental conditions, while configuration may serve as an indicator of the movement and dispersal (i.e., matrix permeability) of species within the surrounding landscape [6,30–32]. Thus, richness, abundance, and turnover of species of leaf-litter ant assemblages have been used as tools for establishing conservation priorities or determining the conservation value of endangered ecosystems, including riparian zones [7,9,23,33].

In this study, performed in a tropical montane cloud forest (TMCF) region, we examined the relative influence of the composition and configuration of the surrounding landscape on the conservation value of riparian vegetation remnants, using leaf-litter ant assemblages as a model group. In general, riparian remnants form part of the most threatened tropical ecosystems worldwide [34], and understanding their function as reservoirs of ant diversity, as determined by the surrounding landscape, has important implications for conservation planning. First, we determined alpha (local) and beta diversity (species turnover) of leaf-litter ants in riparian remnants, and then we assessed if the landscape variables, reflecting different levels of anthropogenic change, influenced ant riparian assemblages.

## Materials and methods

### Ethics statement

Permission to access privately-owned land was given by all land owners. Field collections were carried out under an Ant Collection Permit (SPGA/DGVS/10503/13) issued by the Wildlife Department (Dirección de Vida Silvestre) of Mexico’s Environmental Ministry (SEMARNAT). Due to its focus on invertebrates, this study did not require any approval for animal care and use.

### Study area and site selection

**Fig 1:**
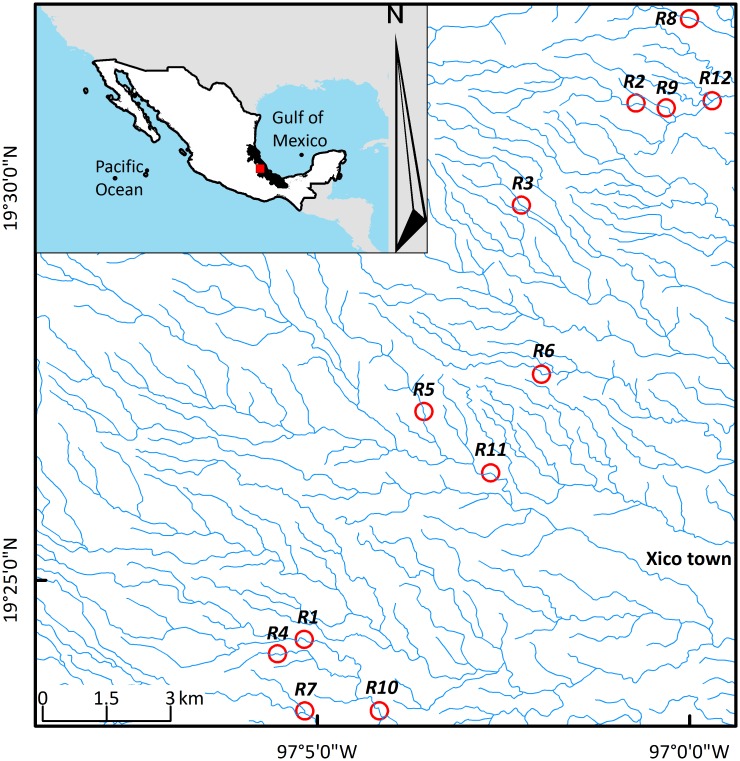
Location of the study sites in central Veracruz, Mexico. The red circles represent 200-m-buffers around each remnant of riparian vegetation where leaf-litter ants were sampled. Blue lines are the tributaries of the Antigua River watershed. In the inset are indicated the location, in Mexico, of the study area (red square), the state of Veracruz (black polygon), and the location of Mexico (white polygon) between North and Central America. This map was generated by the first author (MAGM) using ArcGIS 10.2^®^ and vector data models available in the GIS website of the Mexican commission for the knowledge and use of biodiversity (CONABIO, http://www.conabio.gob.mx/informacion/gis/) under a CC BY license.

The study was conducted in the tropical montane cloud forest region of central Veracruz, Mexico, in the mid-watershed of La Antigua River basin. The climate in the area is mild and humid throughout the year, with total annual precipitation ranging from 1,350 to 2,200 mm and mean annual temperature fluctuating between 12 and 18°C. There are three pronounced seasons: a relatively dry, cool season (October to March), a dry, warm season (April and May), and a wet, warm season (June to September) [34]. In this region, 12 riparian vegetation remnants were selected based on the proportion (range: 5–95%) of forest land cover (i.e., riparian and TMCF land covers) in their surrounding landscape and the accessibility granted by the owners (Fig 1). Patterns of land use and land cover in the watershed and limited access to several zones precluded a balanced design and equidistant sampling. Remnants were located between 1,500 and 2,000 m a.s.l. and separated by a distance ranging from 1 to 18.6 km (Fig 1).

### Landscape characterization

We characterized the landscape surrounding each site using a previously generated LUC map of the study region that belongs to an ongoing project (Castillo *in prep*.). The LUC map was generated from satellite images (SPOT 5; 10 m/pixel) taken on January, 2011. All satellite images were provided by a Mexican satellite receiving station (ERMEX) in 2012. From the LUC map, we created a 200-m buffer from the center of each site. This buffer size was based on previous studies of the effect of landscape on leaf-litter ants [6,31,35]. In order to verify and update the LUC map, we used the most recent satellite imagery available in Google Earth and ground truthing. When necessary, the area within the buffers was corrected, and changes were digitized on-screen with ArcGIS 10.2^®^. The land-use classes in the studied landscape were standardized considering previous studies on the TMCF landscape of this region [34,36,37]. Nine LUC types were defined (Table 1). We used the Patch Analyst extension for ArcMap [38] to compute the spatial metrics of the composition and configuration of the landscape within each buffer of 200 m surrounding the study remnants (S1 Table).

**Table 1:** Land cover types determined from field verification and remotely-sensed data in the studied landscapes of Central Veracruz, Mexico.

Land cover type	Description
Tropical montane cloud forest (TMCF)	Forest fragments with different degrees of disturbance, including secondary forests. Canopy height usually varied between 15 and 30 m and average diameter at breast height (DBH) is greater than 15 cm. The most common species are *Liquidambar styraciflua* L., *Miconia glaberrima* Naudin, *M*. *mexicana* Naudin, *Palicourea padifolia* (Roem. & Schult.), *Quercus germana* Schltdl. & Cham., *Q*. *insignis* M.Martens & Galeotti, *Q*. *laurina* Liebm *Q*. *leiophylla* A.DC., *Q*. *salicifolia* Née, *Q*. *xalapensis* Bonpl., *Turpinia insignis* Tul.
Riparian vegetation	Strips of vegetation immediately adjacent to rivers and streams. Canopy height usually varied between 10 and 25 m and average tree DBH is between 15 and 80 cm. The most common species are *Arachnothryx capitellata* Hemsl., *Buddleja cordata* Kunth, *Meliosma alba* Walp., *Saurauia pedunculata* Hook., *Platanus mexicana* Moric., *Trema micranthum* (L.), *Turpinia occidentalis* G.Don.
Scrub fallow	Second-growth vegetation dominated by shrubs, herbs, and climbing plants. The most common species are *Cestrum nocturnum* L., *Cyphomandra betacea* (Cav.), *Citrus* spp., *Perrottetia longistylis* Rose, *Pipper* spp., *Pteridium aquilinum* (L.), *Rubus* spp., *Smallanthus maculatus* (Cav.) and *Tithonia diversifolia* A.Gray.
Areas reforested with *Pinus*	Different forested areas planted with a single *Pinus* species with a maximum DBH of 12 cm. Management practices include understory clearing and selective logging. Planted species varies widely depending on the provided saplings to farmers by SEMARNAT. At the moment of this study, the most common species were *Pinus leiophylla* Schltdl. & Cham., *P*. *maximinoi* H.E.Moore, *P*. *michoacana* Martínez, *P*. *patula* Schltdl. & Cham., and *P*. *pseudostrobus* Lindl.
Tree crops	Agroforestry systems, including coffee plantations shaded by TMCF species or exotic tree species mainly *Inga* spp.
Shrub crops	Row crops, mainly, of maize, beans, berries, or potato.
Cattle pasture with isolated trees	Active pastures with isolated trees and shrubs. The most common species are *Acacia* spp., *Cedrela odorata* L., *Lippia myriocephala* Schltdl. & Cham. *Psidium guajava* L., and *Randia* spp.
Cattle pasture	Active and intensive pastures usually covered by exotic grasses species like *Andropogon* spp., *Panicum maximum* Jacq., and *Paspalum* spp.
Human settlements and infrastructure	Human populations, cities, or localities, including roads and highways.

We considered riparian remnants to be linear strips of vegetation immediately adjacent to rivers and streams, which widely varied in width and level of impact due to anthropogenic activities [1]. For the landscape composition metrics, we estimated the percentage of land covers with tropical montane cloud forest (TMCF), riparian vegetation, scrub fallow, areas reforested with *Pinus*, tree crops, shrub crops, cattle pasture with isolated trees, cattle pasture, and human settlements and infrastructure within the surrounding landscape (Table 1). In the studied region, some LUCs mimic TMCF cover (e.g., riparian vegetation, scrub fallow, areas reforested with *Pinus*, tree crops), and, when these were contiguous, their edges were not always evident in the observed satellite images. For this reason and in order to verify the interacting LUCs within the 200-m-radius buffers, we did ground truthing to define the natural boundaries among LUCs based on the composition and abundance of plant species (Table 1).

Landscape configuration metrics included the shape and width of each of the 12 focal riparian remnants of this study. The remnant shape was estimated with the shape index proposed by Patton [39]:

$$SI=\frac{P}{\text{2}\cdot \sqrt{\pi \cdot A}}$$ (1)

where *P* and *A* are the patch perimeter and area, respectively. The higher the *SI* values, the higher the shape complexity (perfect circle, *SI* = 1.0). The remnant width was the mean of 10 perpendicular distances, recorded at 10-m intervals along a 100-m transect, between the stream edge and that of the adjoining LUC.

### Ant sampling

Sampling was carried out along a 100-m-long transect in each remnant. Ten 1-m^2^ quadrats of leaf-litter were collected at 10 m intervals along the transect. Five samples were processed in Berlese-Tullgren funnels with a 25-watt light bulb for 72 h, and the other five samples were processed in Winkler sacks for 72 h [40]. These distinct techniques were alternated along the length of each transect. Collections were repeated in both the 2011-rainy and 2012-dry seasons, such that each site had 20 litter samples, 10 in the wet season and 10 in the dry season. All collected ants were preserved in 70% ethanol, and one to five of the collected specimens per sample that differed morphologically were dry-mounted. Only worker ants were counted in the samples and recorded as incidence data for analysis. The Mackay and Mackay [41] key was used to identify ant genera, along with several additional keys for species identification, depending on the genus involved [42–45]. The specimens that could not be identified with the respective keys were identified to morphospecies. All ants, including representative vouchers of each morphospecies, were deposited in the Entomological Collection of the Instituto de Ecología A.C. in Xalapa, Veracruz, Mexico (IEXA; Reg. SEMARNAT: Ver. IN.048.0198).

### Alpha and beta diversity

Number of occurrences of a species at a site, across both wet and dry season sampling, was used as a measure of abundance (with maximum abundance = 20). We calculated the inventory reliability for each remnant using the coverage estimator, which is a less biased estimator of sample completeness than non-parametric methods [46]:

$$\hat{C}n=\left(1–\frac{f_{1}}{n}\left[\frac{\text{(}n-1)f_{\text{1}}}{\left(n-1\right)f_{1}+2f_{2}}\right]\right)*100$$ (2)

where *n* is the number of occurrences summed across all species of a given site, and *f*_*1*_ and *f*_*2*_ are singletons (species each represented by only a single occurrence) and doubletons (species each represented by exactly two occurrences), respectively. This sample completeness (*Ĉn*) indicates the proportion of the ‘total community’ represented by the trapped species [46] and enables comparison of the diversity of assemblages at the same sample coverage [46,47]. When *Ĉn* ≈ 100%, sampling is complete given the effort and capture technique used [46]. Values of *Ĉn* were calculated using iNEXT package for R [48].

Ant diversity was evaluated using Hill numbers [49], following Jost’s [50] proposal. These measures are recommended for comparative studies of diversity because they meet the replication principle [51] and are easy to interpret [47]. We used Hill numbers of order 0 (^*0*^*D*, species richness), 1 (^*1*^*D*, exponential of Shannon’s entropy), and 2 (^*2*^*D*, inverse Simpson concentration). Species richness (^*0*^*D*) is not sensitive to species abundances and thus gives disproportionate weight to rare species [50]. Shannon diversity (^*1*^*D*) weighs each species according to its abundance in the community; hence, it can be interpreted as the number of ‘common’ or ‘typical’ species in the community [50]. Finally, Simpson diversity (^*2*^*D*) can be interpreted as the number of ‘very abundant’ or ‘dominant’ species in the community [50]. To compare each diversity measure among remnants, we used 95% confidence intervals in which significant differences were indicated by non-overlapping confidence intervals [52].

To evaluate differences in species’ dominance, rarity, and community evenness among study remnants and thus better interpret our results, ant abundance was represented by rank-abundance species curves or Whittaker plots [53]. We plotted the proportional abundance of each species, ordered from the most to the least abundant, in order to show differences in species’ dominance and rarity in addition to the assemblage evenness among remnants.

For analyzing beta diversity, we determined the compositional similarity among assemblages using the indices of Jaccard, Sørensen, and Morisita-Horn. These indices have values ranging from 0 (minimal similarity) to 100 (maximum similarity) [53]. We used these indices because, as with the Hill numbers, each provides distinct information about compositional similarity depending on their sensitivity to species’ abundances (i.e., sensitivity to rare or common species) [50]. The Jaccard index only takes into account shared species and presence/absence of species between sites [50]. The Sørensen index relates the sum of the lower of the two abundances for shared species with the total abundance observed between sites [53]. The Morisita-Horn index relates the abundance of each species with the abundance of the most abundant species between sites [53]. In a unified framework of analysis under a scheme of diversity measured as the effective number of species, the Jaccard, Sørensen, and Morisita-Horn indices represent simple monotonic transformations of the beta diversity of orders 0 (^*0*^*D*_*β*_), 1 (^*1*^*D*_*β*_), and 2 (^*2*^*D*_*β*_), respectively. In other words, the beta diversity of orders 0 (^*0*^*D*_*β*_), 1 (^*1*^*D*_*β*_), and 2 (^*2*^*D*_*β*_) are inversely related to the Jaccard, Sørensen, and Morisita-Horn indices of compositional similarity, respectively (i.e., if the communities have a high compositional similarity, then the set of communities must have a low beta diversity) [50].

The compositional similarity among remnants was represented by a cluster analysis using the Unweighted Pair Group Method with Arithmetic Mean (UPGMA) linkage technique. For *post hoc* analyses, similarity profile tests (SIMPROF) were used as statistical tests to compare similarity among assemblages in the PRIMER program version 6.1.16 [54]. SIMPROF test assumes that a real clustering of assemblages will be evidenced by an excess of smaller and/or larger similarities than expected under the null hypothesis that all assemblages are drawn from the same species assemblage [55].

### Landscape predictors

In order to identify the dominant landscape predictors influencing alpha and beta ant diversity of riparian remnants, we followed both univariate and multivariate selection procedures for regression-based models. As these statistical techniques are sensitive to collinearity between predictor variables, we used the Spearman correlation coefficient to exclude correlated variables. For each set of significantly correlated variables we retained only one that was considered to be the most intuitive and interpretable.

We used generalized linear models (GLM) to assess the independent effects of each landscape predictor on each metric of alpha diversity (i.e., a single univariate regression between a response and a predictor variable). We applied a Gaussian error distribution for continuous variables (i.e., species richness, Shannon and Simpson diversity) after testing for normality (Shapiro–Wilk test). Abundance (a count-dependent variable) was assessed assuming a Poisson error distribution. For each multiple regression model (i.e., a multiple univariate regression between a response and several predictor variables), we used the variance inflation factors (VIF) to exclude landscape predictors that would affect the accuracy of the estimates, using the *car* package for R version 3.2.2. We followed an information-theoretic approach and multi-model inference to assess the relative effect of each landscape predictor on each metric of alpha diversity using the package *glmulti* for R version 3.2.2 [56,57]. This function built a set of models representing all possible combinations of landscape predictors for each diversity measure. It also computed the Akaike’s information criterion, corrected for small samples (AICc) for each built model. To correct for the overdispersion associated with count data, abundance was assessed with qAICc instead of AICc values [57]. The goodness-of-fit of the models was estimated as the explained deviance for each complete model using the *modEvA* package for R version 3.2.2 [58].

We used distance-based linear models (DistLM) for analyzing and modelling the relationships between the distance/similarity matrices of ant assemblages and the landscape predictors. Using a multiple regression model, this routine performed a partition according to the variation in the data cloud that was described by the resemblance matrices. Then, it performed a permutational test for the multivariate null hypothesis that no relationship existed between explanatory and response matrices, based on a chosen resemblance measure and using permutations of the samples to obtain a P-value. Finally, it modeled the percentage of overall variation in the compositional similarity of ant assemblages accounted for by each landscape predictor [59]. In this procedure we considered the compositional similarity between assemblages as response matrices, using the Jaccard (^*0*^*D*_*β*_), Sørensen (^*1*^*D*_*β*_), and Morisita-Horn (^*2*^*D*_*β*_) indices. The Akaike’s Information Criterion for small samples (AICc) was tested in the analysis to provide a comprehensive evaluation of appropriate predictors to include in the models. The selection procedure of the models was “BEST”, which examines the value of the selection criterion for all possible combinations of predictor variables. These analyses were carried out using PRIMER ver. 6.1.18 and PERMANOVA+ ver. 1.0.8 [59,60].

Following Burnham and Anderson [56], we considered a set of models with equivalently strong empirical support and similar plausibility, or when the difference in the qAICc or AICc values were less than 2 in comparison to the best model (i.e., the one with lowest qAICc or AICc value). To evaluate the importance of each predictor and to produce model-averaged parameter estimates, we used Akaike weights (*w*_*i*_), which represent the probability that a particular model would be selected as the best fitting model if the data were to be collected again under identical conditions. This model can therefore be considered as the best model for a particular dataset. Thus, we summed *w*_*i*_ of ranked models until the total was > 0.95. The set of models for which a sum of *w*_*i*_ was 0.95 represented a set that had a 95%-probability of containing the true best model. The relative importance of each predictor was assessed based on the sum of Akaike weights (*∑w*_*i*_) of each candidate model in which the predictor appeared. We considered a given landscape predictor to be an important explanatory variable for a given diversity measure when it showed a high sum of Akaike weights (i.e., considering each candidate model in which it appeared) and when its model-averaged unconditional variance was lower than the model-averaged parameter estimate [56].

In order to examine whether the proximity in remnants or buffers of surrounding landscape (Fig 1) influenced the GLM or DistLM regressions, we tested for spatial autocorrelation in the landscape predictors [61]. We examined the degree of spatial autocorrelation in the residuals of the GLM regressions with the Moran’s test for spatial autocorrelation using a spatial weights matrix in the *spdep* package for R version 3.2.2 [62]. For the calculation of Moran’s I, we used nearest neighbor distances as the metric and the permutation test option. For examining the spatial autocorrelation in the residuals of the DistLM multivariate regressions, we performed a multivariate spatial Mantel analysis using the *MRM* function in the *ecodist* package for R version 3.2.2 [63,64]. None of the variables examined for the GLM or DistLM regressions displayed significant spatial autocorrelation at any distance (S2 Table).

## Results

### Landscape patterns

In the studied landscapes, riparian land cover varied from 6% (R2) to 66% (R9), and it was negatively correlated with the land covers with areas reforested with *Pinus* (ρ = -0.71, P < 0.05), cattle pastures (ρ = -0.88, P < 0.05), and human settlements and infrastructure (ρ = -0.74, P < 0.05, S1 Table). TMCF land cover varied from 7% (R1) to 45% (R12), cattle pastures with isolated trees from 0% (R9) to 28% (R4), and scrub fallow from 0% (R1, R10, R11, and R12) to 28% (R6). The highest proportion of tree crop land cover was observed in R8 (10%), followed by R3 (1%), R4, and R5 (less than 1% each), and the remaining had 0%. The highest shrub crop land cover was found in R3 and R4 (4% each), followed by R2 and R7 (2%), R6 (1%), R5, and R10 (less than 1% each), and the remaining had 0%. The percentage of land covers with TMCF, scrub fallow, tree crops, shrub crops, and cattle pasture with isolated trees within the surrounding landscape were not significantly correlated among them or to any other land cover (P > 0.05).

### Alpha and beta diversities

We collected 8,684 individuals belonging to 53 species, 22 genera, 11 tribes, and 7 subfamilies (S3 Table). Subfamily Myrmicinae had the highest number of tribes, genera, and species. The richest genus was *Stenamma* (7 spp.), followed by *Adelomyrmex* (5 spp.), *Hypoponera*, *Nylanderia*, and *Pheidole* (5 spp. each), *Solenopsis* and *Strumigenys* (3 spp. each), and *Brachymyrmex*, *Eurhopalothrix*, *Gnamptogenys*, *Labidus*, and *Temnothorax* (2 spp. each). The 10 remaining genera were represented by only one species.

The average sample coverage was 97% (range: 92–98%). The overall sample coverage, considering the 12 riparian remnants, was 99% (S4 Table). Abundance varied between 83 (R11) and 118 (R3) species occurrences, and the assemblage structure changed across the sampled remnants (S1 Fig). The general pattern observed indicated a decrease in species dominance from R1 to R12, and the dominant species were different in each remnant. Species richness (^*0*^*D*) varied significantly from 9 (R1 and R2) to 26 (R12) species. The diversity of Shannon (^*1*^*D*) increased significantly from R2 (8 spp.) to R12 (23 spp.), and that of the order 2 (^*2*^*D*) increased significantly from R4 (7 spp.) to R11 and R12 (19 spp.) (S4 Table).

The compositional similarity using cluster analysis and SIMPROF tests indicated that the Jaccard index significantly separated three assemblage clusters with similarities of 34% (*π* = 3.27, P = 0.001) and 40% (*π* = 3.19, P = 0.006, S2 Fig). The Sørensen index significantly separated two assemblage clusters at a similarity of 33% (*π* = 4.5, P = 0.001, S2 Fig). Meanwhile, the Morisita-Horn index significantly separated six assemblage clusters at similarities of 42% (π = 3.7, P = 0.001), 53% (π = 3.19, P = 0.01), 66% (*π* = 6.27, P = 0.001), 70% (*π* = 5.87, P = 0.01), and 73% (*π* = 8.92, P = 0.007, S2 Fig).

### Landscape predictors of alpha and beta diversity

**Fig 2:**
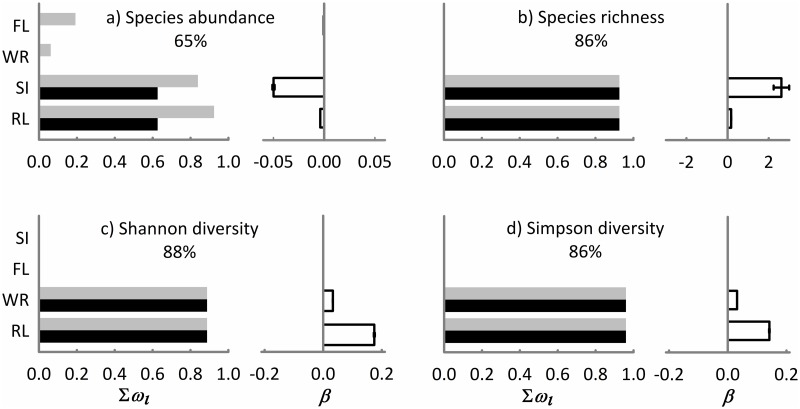
Landscape predictors included in the 95% confidence set of the models (gray bars) and in the ΔAICc < 2 set of the models (black bars) for explaining the abundance, species richness, Shannon, and Simpson diversity of leaf-litter ant assemblages associated with remnants of riparian vegetation in central Veracruz, Mexico. The importance of each predictor is shown by the sum of Akaike weights (*∑w*_*i*_, panels in the left side). Panels on the right side indicate the values of the averaged model parameter estimates (*β*) ± unconditional variance of information-theory-based model selection and multi-model inference. The sign (±) of parameter estimates represents a positive or negative effect of the predictor on the diversity measures. The goodness-of-fit of each multiple model is indicated in each panel as the percentage of deviance explained by each multiple model. The predictors are the percentage of riparian land cover (RL) and tropical montane cloud forest land cover (FL) within the surrounding landscape, the shape (SI) and width (WR) of the focal riparian remnant.

Riparian land cover in the landscape was one of the most important predictors that explained abundance, richness, and diversity of species. TMCF land cover was only significantly related with ant abundance. Meanwhile, land covers with cattle pastures with isolated trees, scrub fallow, shrub crops, and tree crops were not significantly related with any abundance or diversity variable. The multiple models explained between 65 and 88% of the deviance (Fig 2).

GLM analyses indicated that abundance was negatively (and independently) explained by the riparian and TMCF land covers in the landscape as well as by the shape and width of the focal riparian remnant (Table 2). The information-theoretic approach and multi-model inference analysis indicated that in a multiple model, riparian land cover and shape of the focal remnants were the most important predictors of abundance (Fig 2a). Species richness was positively (and independently) explained by riparian land cover and the shape and width of the focal riparian remnants (Table 2). In a multiple model, riparian land cover and shape of the focal remnant were the most important predictors of species richness (Fig 2b). Shannon diversity was positively (and independently) explained by riparian land cover and width of the focal riparian remnants (Table 2). In a multiple model, riparian land cover and shape of the focal remnant were equally important predictors of Shannon diversity (Fig 2c). Simpson diversity was positively (and independently) explained by riparian land cover and the width of the focal riparian remnant (Table 2). In a multiple model, riparian land cover and shape of the focal remnants were equally important predictors of Shannon diversity (Fig 2d).

**Table 2:** Effects of the landscape characteristics on alpha and beta diversity of leaf-litter ants associated with remnants of riparian vegetation. Relationships between landscape predictors and alpha diversity metrics and between landscape predictors and beta diversity metrics are indicated separately.

**A) Single and univariate generalized linear models**	***Z* or *t* statistic**	**d.f.**	***P***	**AICc**
Abundance ~ Riparian land cover	-3.63	10	0.0002	188.39
Abundance ~ TMCF land cover	-2.4	10	0.0100	205.26
Abundance ~ Shape of focal remnant	-3.41	10	0.0006	192.45
Abundance ~ Width of focal remnant	-3.07	10	0.0020	196.91
Species richness ~ Riparian land cover	4.82	10	0.0006	71.01
Species richness ~ TMCF land cover	1.54	10	0.1500	81.77
Species richness ~ Shape of focal remnant	3.87	10	0.0030	74.42
Species richness ~ Width of focal remnant	3.85	10	0.0030	74.52
Shannon diversity ~ Riparian land cover	8.33	10	<0.0001	57.99
Shannon diversity ~ TMCF land cover	1.82	10	0.0986	76.42
Shannon diversity ~ Shape of focal remnant	2.20	10	0.0523	75.11
Shannon diversity ~ Width of focal remnant	6.30	10	<0.0001	63.59
Simpson diversity ~ Riparian land cover	8.54	10	<0.0001	53.76
Simpson diversity ~ TMCF land cover	1.80	10	0.1020	72.80
Simpson diversity ~ Shape of focal remnant	1.88	10	0.0889	72.52
Simpson diversity ~ Width of focal remnant	6.70	10	<0.0001	58.73
**B) Multiple and univariate generalized linear models**	***Z* or *t* statistic**	**d.f.**	***P***	**AICc**
Abundance ~				
Riparian land cover	-2.50	10	0.0125	188.39
+ Shape of focal remnant	-2.01	9	0.0449	183.86
Species richness ~				
Riparian land cover	5.50	10	0.0004	71.01
+ Shape of focal remnant	4.53	9	0.0014	61.46
Shannon diversity ~				
Riparian land cover	4.62	10	0.0013	57.99
+ Width of focal remnant	3.16	9	0.0115	53.73
Simpson diversity ~				
Riparian land cover +	5.072	10	0.0007	53.76
+ Width of focal remnant	3.734	9	0.0047	47.24
**C) Single and multivariate distance-based linear models**	***Pseudo-F***	**d.f.**	***P***	**AICc**
Jaccard similarity ~ Riparian land cover	3.55	1	0.0027	91.06
Jaccard similarity ~ TMCF land cover	2.23	1	0.0369	92.29
Jaccard similarity ~ Shape of focal remnant	2.75	1	0.0120	91.79
Jaccard similarity ~ Width of focal remnant	3.54	1	0.0032	91.07
Sørensen similarity ~ Riparian land cover	6.03	1	0.0008	87.92
Sørensen similarity ~ TMCF land cover	2.20	1	0.0718	91.20
Sørensen similarity ~ Shape of focal remnant	3.03	1	0.0283	90.41
Sørensen similarity ~ Width of focal remnant	6.18	1	0.0011	87.81
Morisita-Horn similarity ~ Riparian land cover	7.47	1	0.0018	83.04
Morisita-Horn similarity ~ TMCF land cover	2.56	1	0.0821	87.00
Morisita-Horn similarity ~ Shape of focal remnant	4.01	1	0.0243	85.69
Morisita-Horn similarity ~ Width of focal remnant	10.26	1	0.0003	81.26
**D) Multiple and multivariate distance-based linear models**	***Pseudo-F***	**d.f.**	***P***	**AICc**
Jaccard similarity ~				
Riparian land cover	9.97	1	0.0004	91.06
+ Width of focal remnant	3.91	1	0.0003	92.83
+ Shape of focal remnant	2.36	1	0.0207	95.09
Sørensen similarity ~				
Riparian land cover	6.02	1	0.0008	87.92
+ Width of focal remnant	6.17	1	0.0004	89.39
+ Shape of focal remnant	3.03	1	0.0291	91.89
Morisita-Horn similarity ~				
Riparian land cover	7.46	1	0.0023	83.04
+ Width of focal remnant	10.26	1	0.0005	83.71
+ Shape of focal remnant	4.01	1	0.0252	86.22

**Fig 3:**
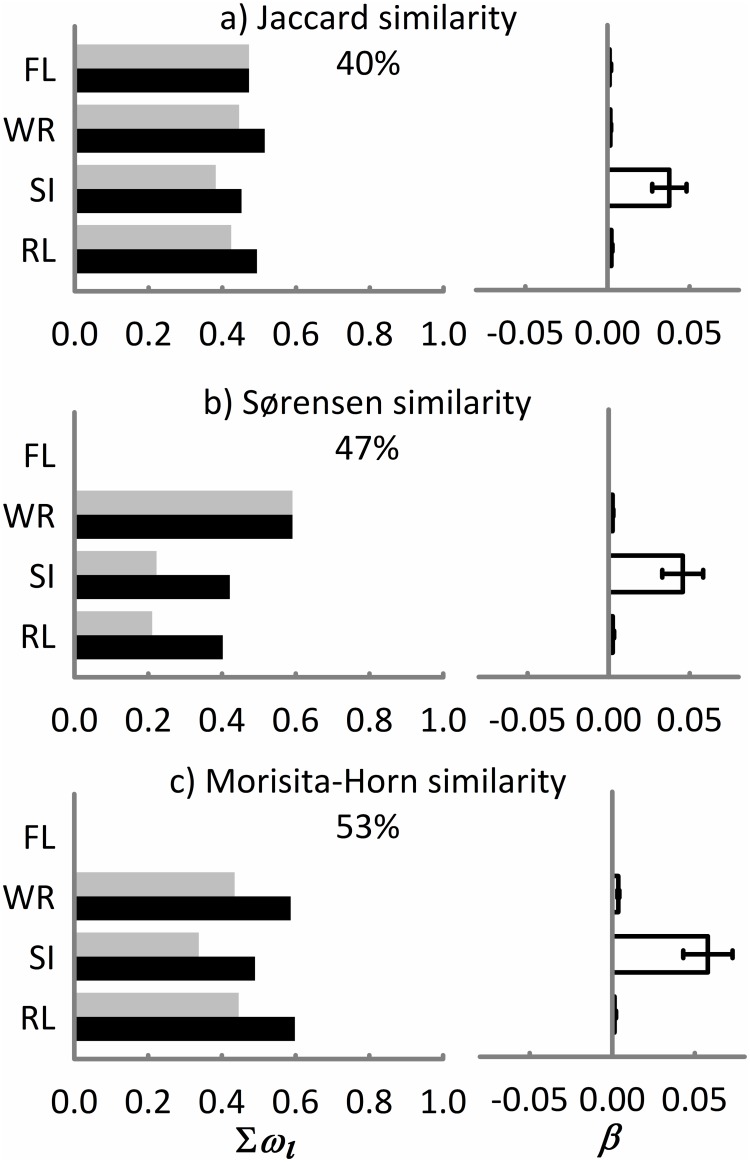
Landscape predictors included in the 95% confidence set of the models (gray bars) and in the ΔAICc < 2 set of the models (black bars) for explaining the compositional similarity indicated by Jaccard, Sørensen, and Morisita-Horn indices of leaf-litter ant assemblages associated with remnants of riparian vegetation in central Veracruz, Mexico. The importance of each predictor is shown by the sum of Akaike weights (*∑w*_*i*_, panels in the left side). Panels on the right side indicate the values of the averaged model parameter estimates (*β*) ± unconditional variance of information-theory-based model selection and multi-model inference. The sign (±) of parameter estimates represents a positive or negative effect of the predictor on the diversity measures. The goodness-of-fit of each multiple model is indicated in each panel as the percentage of total variation explained by each multiple model. The predictors are the percentage of riparian land cover (RL) and tropical montane cloud forest land cover (FL) within the surrounding landscape, the shape (SI) and width (WR) of the focal riparian remnant.

The distance-based linear modelling (DistLM) indicated that species composition (Jaccard index, ^*0*^*D*_*β*_) was independently explained by the riparian and TMCF land covers in the landscape as well as by the shape and width of the focal riparian remnant (Table 2). The information-theoretic approach and multi-model inference analysis indicated that in a multiple model, the riparian land cover within the landscape and the width of the focal riparian remnant were the most important predictors of Jaccard similarity (Fig 3a). Compositional similarity of order 1 (^*1*^*D*_*β*_, Sørensen index) was independently explained by the riparian land cover in the landscape and the shape and width of the focal riparian remnant (Table 2). In a multiple model, the width of the focal riparian remnants was the most important predictor of Sørensen similarity (Fig 3b). Compositional similarity of order 2 (^*2*^*D*_*β*_, Morisita-Horn index) was independently explained by the riparian land cover in the landscape and by the shape and width of the focal riparian remnants (Table 2). In a multiple model, the riparian land cover within the landscape and the width of the focal riparian remnant were the most important predictors of Morisita-Horn similarity (Fig 3c).

## Discussion

This study supports the importance of variables at the landscape level and their effect on the alpha and beta diversity of leaf-litter ants associated with riparian vegetation remnants in the TMCF region of central Veracruz, Mexico. Our results also improve the understanding of the main drivers determining the riparian assemblages of leaf-litter ants in fragmented tropical montane landscapes. Overall, in the studied landscape alpha diversity metrics and compositional similarity were mainly shaped by the extent of riparian land cover and the width of riparian remnants.

In general, species richness, Shannon and Simpson diversity increased significantly in remnants within landscapes with a high percentage of riparian land cover and a low percentage of land covers with areas reforested with *Pinus*, cattle pastures, and human settlements and infrastructure (S1 and S4 Tables, Fig 2). These results are consistent with other studies that also found that ant diversity is positively related to the amount of remaining natural habitat in the landscape [6,8,30,31]. This is not surprising, as this ecological group is expected to be vulnerable to changes in the amount of available habitat [32]. With an increase in riparian land cover, we could expect a greater potential availability and quality of nesting sites, in addition to a larger supply of food, as well as favorable environmental conditions that would support richer leaf-litter ant assemblages [3,8–10,32,65].

The observed diversity pattern may additionally be related to the heterogeneity of the studied landscape, where several small riparian remnants were more isolated from each other in comparison to a few large and less isolated remnants (S1 Table). Although our results indicate that riparian land cover in the surrounding landscape is the main driver of species diversity, the shape and width of focal riparian remnants were also important predictors (Fig 2). We found that the width of focal riparian remnants was an important predictor positively (and significantly) related to increases in Shannon and Simpson diversity. Meanwhile, the shape of remnants was significantly related to increases in species richness and decreases in abundance of leaf-litter assemblages. Commonly, a high edge to area ratio increases species loss, and Didham [19] suggests that this effect is likely to be particularly severe for remnants of riparian vegetation. Surprisingly, we found contradictory results for leaf-litter ants. Even so, shape complexity is, until now, a landscape attribute that has not been well studied [19,66]. Patch shape complexity has been highlighted as influential in the extent to which edge effects permeate habitat patches and reduce core area for patch specialists [66]. In this study, the significance of riparian remnants with an irregular shape may be that they counterbalance the loss of species diversity due to spillover or the active movement of leaf-litter ant species from the surrounding land covers [67]. In this sense, riparian remnants could act as supplementary or complementary habitats and offer various resources to a species pool of leaf-litter ants that cannot distinguish between habitat and matrix [19]. The species that move to riparian remnants or use them in some way may differ depending on the surrounding landscape composition [67]. For example, in landscapes with remnants that are wider and more complex in shape, we collected cryptic and specialist species reported for TMCF (e.g., *Adelomyrmex* spp., *Eurhopalothrix* spp., *Stenamma* spp., and *Strumigenys* spp.; S3 Table) [24]. In contrast, in landscapes composed of narrow and less complex riparian remnants, we found generalist species that are common in open areas and tolerant to these conditions (e.g. *Brachymyrmex* spp., *Nylanderia* spp., and *Solenopsis* spp.; S3 Table) [13].

In the comparison of compositional similarity, we found from 2 (Sørensen index) to 6 (Morisita-Horn index) significant clusterings or effective communities *sensu* Jost [50] of leaf-litter ant assemblages (S2 Fig). These results indicate that ant assemblages become more different when abundant species are considered in the similarity composition. This pattern of differentiation in composition may be explained by the relatively high fraction of unique remnant species (34% of the total collected species) and low fraction of numerically dominant species (7%, S1 Table, S1 and S2 Figs). This result has been previously shown for leaf-litter ant assemblages associated with TMCF fragments and cattle pastures with isolated trees in the studied region [3,13,24]. Therefore, this high species turnover among remnants suggests that the maintenance of even highly disturbed riparian remnants may play a strategic role in the conservation of myrmecofauna and probably of other organisms in the severely transformed landscape of this region.

The observed compositional similarity of ant assemblages is likely a function of the percentage of riparian land cover in the surrounding landscape (Table 2, Fig 3). Some studies have suggested that there is a general pattern of differentiation in the compositional similarity that corresponds with changes in the configuration and composition of the surrounding landscape [32]. In particular, our results are consistent with previous studies suggesting that shifts in species composition are attributable to variations in the proportion of available habitat and the land cover types present in the surrounding landscape, mainly when there are large extensions of open areas like cattle pastures [68,69]. We observed in the studied landscape that certain surrounding land uses, such as pine plantations and human settlements or infrastructure, are the main threats to riparian land cover (S1 Table). That kind of surrounding landscape was also reported as an important driver of compositional similarity for ants in a sandhill habitat in Florida, USA [30]. In our study, these land uses led to a replacement of specialist ant species by generalists within riparian remnants. Additionally, that surrounding landscape plays an important role in structuring ant assemblages via influencing the dynamic of colonization-extinction and limiting the dispersal of communities across the fragmented region [30]. Thus, at the landscape scale, the composition of the surrounding landscape may explain the variation in compositional similarity among riparian assemblages (S1 Table, Fig 3).

In conclusion, this study found evidence that the diversity of leaf-litter ants, a highly specialized guild of arthropods, is significantly impacted by both composition and configuration of the surrounding landscape. At the small landscape-scale (200-m-buffers), considering nine land cover types in a highly transformed landscape, the extent of the riparian land cover within the surrounding landscape determined the capacity of riparian remnants to conserve ants. Based on our results and the bioindicator capacity of leaf-litter ant assemblages, maintaining the remaining riparian remnants could be a viable strategy to conserve biodiversity and environmental services in the study region (Fig 1). Conservation actions should involve the active protection and restoration of native forest (TMCF and riparian vegetation) in order to increase the permeability of the surrounding landscape at small scales. That strategy may result in a positive impact on biodiversity conservation. Viable alternatives to reconcile conservation and land productivity should be explored (forest-pastoral systems, expansion of riparian vegetation with useful native tree plantations, enrichment of pine plantations, etc.) [70,71].

Many studies have investigated the optimal strategies in riparian zones for conserving a wide range of taxa, including amphibians, reptiles, birds, mammals, and plants [11,14–16,72]. However, data on invertebrates are still limited [6,8]. The observed results for the studied bioindicator group suggest that policies and strategies that take into account habitat-level features in order to improve the conservation value of riparian remnants should also consider features of the surrounding landscape. In particular, riparian remnants are highly influenced by their surroundings, and increasing forested areas in the surrounding landscape, as well as the width and heterogeneous shape of riparian remnants, will stimulate biodiversity movement. In addition, such a strategy could foster and protect the ecosystem services offered by the forest and riparian vegetation in the studied landscape [34]. Finally, effective outcomes will only be achieved if scientific knowledge is considered during the early planning stages of policies that affect riparian zones, in addition to the subsequent integration of riparian policies into broader environmental planning instruments [8].

## Supporting information

S1 Fig Rank-abundance curves of the leaf-litter ant assemblages associated with remnants of riparian vegetation in central Veracruz, Mexico. Only species with a relative abundance higher than 5% (above dashed line) in a given remnant are shown. Ant species are numbered in accordance with S2 Table.
(TIF)

S2 Fig Dendrograms from standard hierarchical clustering based on the Jaccard (^*0*^*D*_*β*_), Sørensen (^*1*^*D*_*β*_), and Morisita-Horn (^*2*^*D*_*β*_) similarity indices of the leaf-litter ant assemblages associated with remnants of riparian vegetation. The dendrogram displays with black continuous lines the divisions for which the SIMPROF test rejects the null hypothesis (where assemblages in that group have no further structure to explore) and with red dashed lines the groups of assemblages not separated (at P < 0.05) by SIMPROF.
(TIF)

S1 Table Landscape metrics for all twelve remnants of riparian vegetation in central Veracruz, Mexico. (XLSX)

S2 Table Results from spatial autocorrelation of the landscape predictors to examine whether the proximity in remnants or buffers of surrounding landscape influenced the regressions of generalized linear models (A and B) or multivariate distance-based linear models (C and D). (XLSX)

S3 Table Leaf-litter ants collected in each remnant of riparian vegetation in the central mountainous region of Veracruz, Mexico. All species are sorted by subfamily and tribe. Number listed indicate the observed species occurrences in each site.
(XLSX)

S4 Table Sampling completeness and alpha diversity of the leaf-litter ants associated with 12 remnants of riparian vegetation in central Veracruz, Mexico. The lower and upper 95% confidence intervals for each diversity measure are given in brackets. Numbers listed as abundance indicate the sum of all species occurrences per remnant during both dry and wet seasons.
(DOCX)
