# Supplementary figures and images for: The surrounding landscape influences the diversity of leaf-litter ants in riparian cloud forest remnants
Source: PLoS One. 2017 Feb 24;12(2):e0172464. doi: 10.1371/journal.pone.0172464 (PMC5325296; doi:10.1371/journal.pone.0172464)

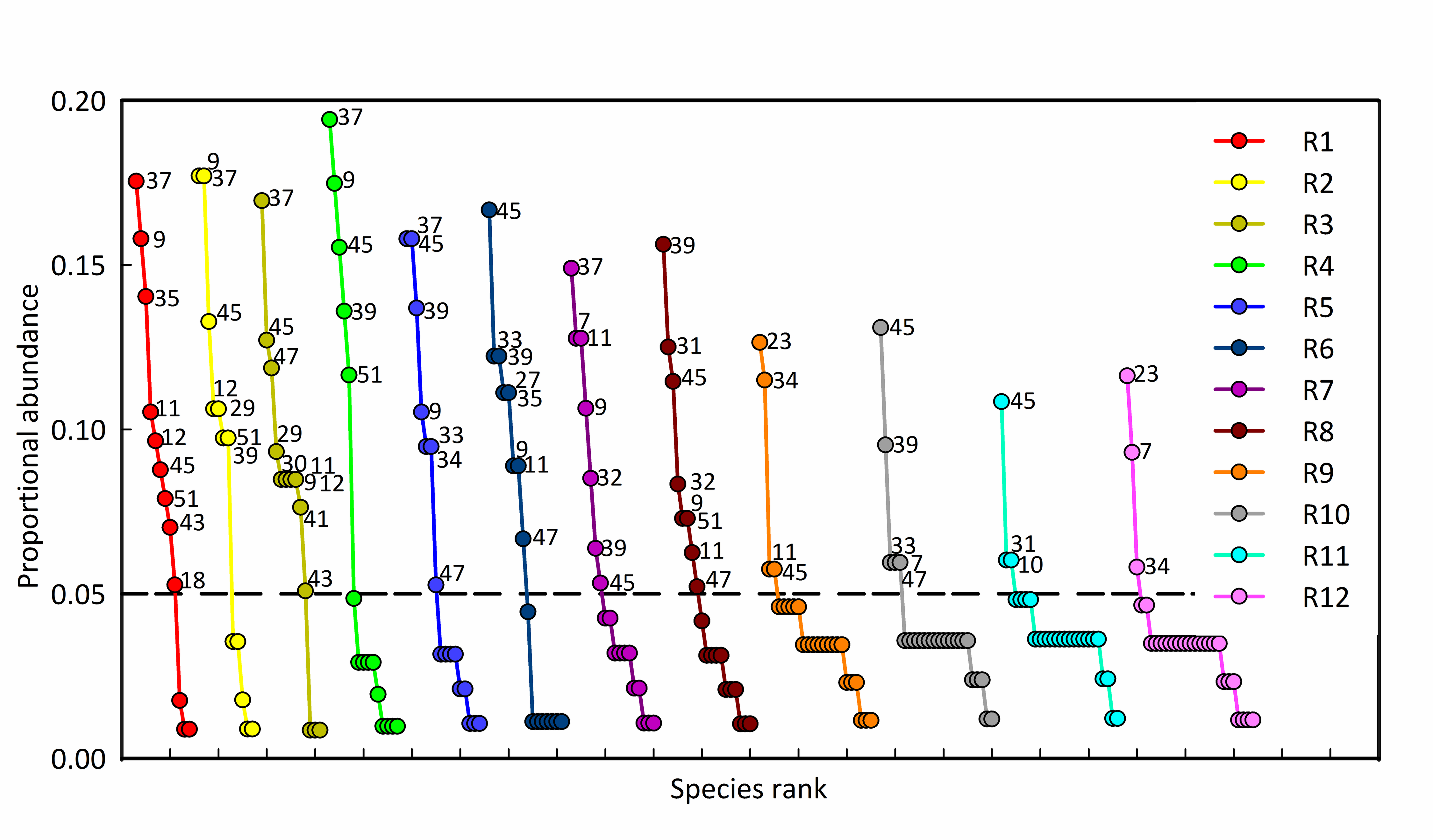

Supplement: S1 Fig — Only species with a relative abundance higher than 5% (above dashed line) in a given remnant are shown. Ant species are numbered in accordance with S2 Table. (TIF) [file pone.0172464.s001.tif]

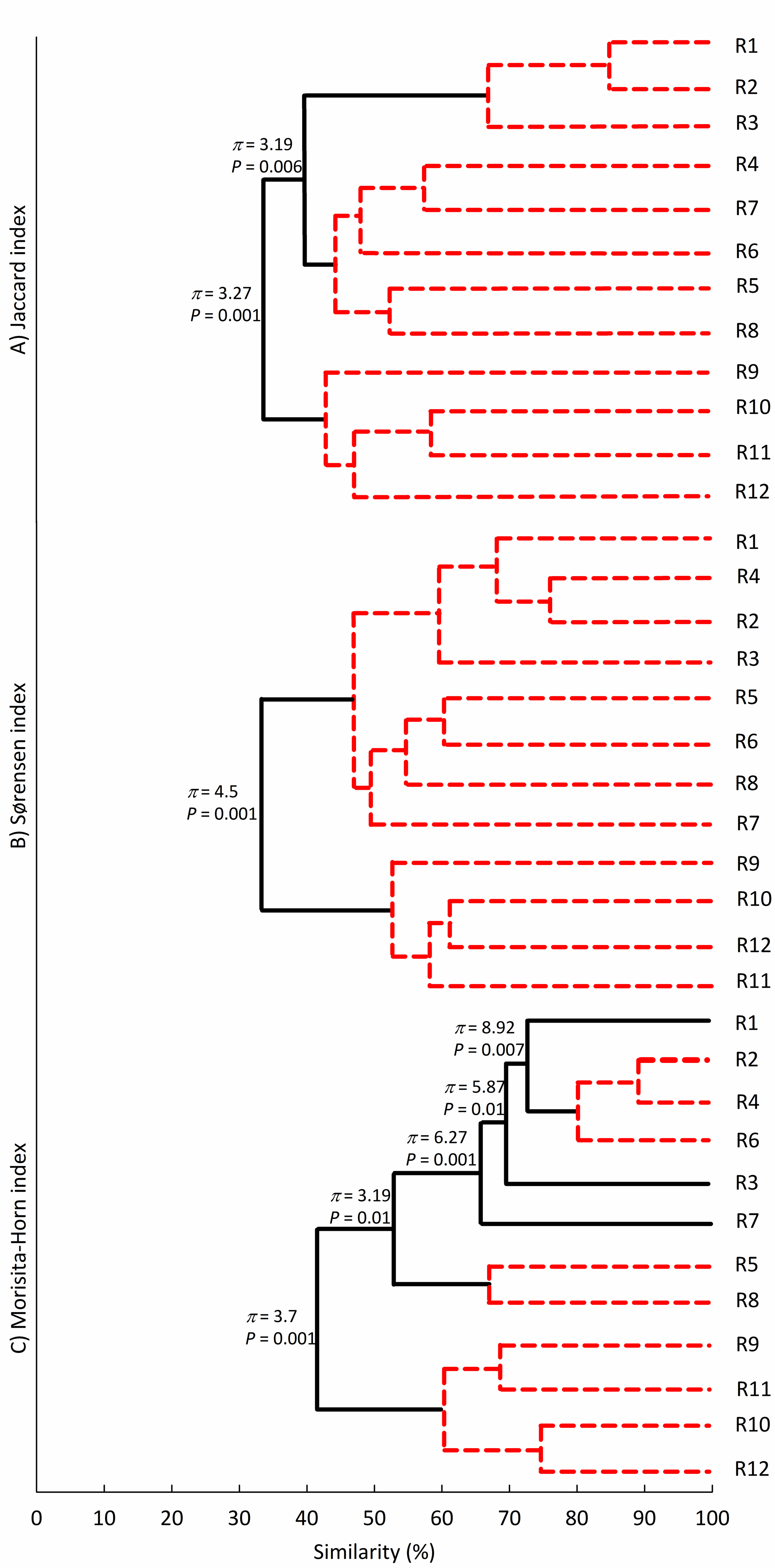

Supplement: S2 Fig — The dendrogram displays with black continuous lines the divisions for which the SIMPROF test rejects the null hypothesis (where assemblages in that group have no further structure to explore) and with red dashed lines the groups of assemblages not separated (at P < 0.05) by SIMPROF. (TIF) [file pone.0172464.s002.tif]
