# Supplementary material for: The surrounding landscape influences the diversity of leaf-litter ants in riparian cloud forest remnants
Source: PLoS One. 2017 Feb 24;12(2):e0172464. doi: 10.1371/journal.pone.0172464 (PMC5325296; doi:10.1371/journal.pone.0172464)
Supplement: S4 Table — The lower and upper 95% confidence intervals for each diversity measure are given in brackets. Numbers listed as abundance indicate the sum of all species occurrences per remnant during both dry and wet seasons. (DOCX) [file pone.0172464.s006.docx]

**S4 Table. Sampling completeness and alpha diversity of the leaf-litter ants associated to 12 remnants of riparian vegetation in central Veracruz, Mexico.** The lower and upper 95% confidence intervals for each diversity measure are given in brackets. Numbers listed as abundance indicate the sum of all species occurrences per remnant across both wet and dry season sampling.

| **Remnant** | **Observed Sampling Completeness** | **Observed species richness** | **Abundance** | **Species richness (*^0^D*)** | **Shannon diversity (*^1^D*)** | **Simpson diversity (*^2^D*)** |
| --- | --- | --- | --- | --- | --- | --- |
| R1 | 98.3 (96.6-100) | 12 | 114 | 9.71 (8.94-10.48) | 8.58 (7.79-9.38) | 7.88 (7.14-8.61) |
| R2 | 98.3 (96.3-100) | 12 | 113 | 9.99 (8.86-11.13) | 8.49 (7.66-9.32) | 7.7 (6.99-8.41) |
| R3 | 97.6 (95.6-99.6) | 13 | 118 | 10.52 (9.64-11.39) | 9.42 (8.67-10.17) | 8.68 (7.98-9.39) |
| R4 | 96.2 (92.7-99.7) | 15 | 103 | 13.91 (11.23-16.6) | 9.27 (7.9-10.64) | 7.57 (6.51-8.64) |
| R5 | 92.4 (87.7-97) | 16 | 95 | 21.86 (13.86-29.86) | 11.9 (9.7-14.1) | 9.77 (8.49-11.05) |
| R6 | 97 (93.6-100) | 16 | 90 | 14.87 (12.63-17.11) | 11.05 (9.68-12.41) | 9.26 (8.1-10.43) |
| R7 | 97 (92.9-100) | 18 | 94 | 17.11 (14.83-19.39) | 13.13 (11.33-14.94) | 10.95 (9.29-12.61) |
| R8 | 97.2 (93.7-100) | 19 | 96 | 18.06 (15.78-20.33) | 13.9 (12.11-15.69) | 11.52 (9.91-13.13) |
| R9 | 97.9 (93.1-100) | 24 | 87 | 23.46 (21.12-25.79) | 19.99 (17.57-22.41) | 16.93 (14.31-19.54) |
| R10 | 96.9 (93.5-100) | 24 | 84 | 23.5 (21.04-25.95) | 19.69 (16.58-22.79) | 16.49 (12.93-20.05) |
| R11 | 97.8 (92.9-100) | 25 | 83 | 24.49 (22.41-26.57) | 21.99 (19.66-24.33) | 19.77 (17.04-22.5) |
| R12 | 95.7 (90.6-100) | 27 | 86 | 26.8 (24.38-29.22) | 23.07 (19.96-26.17) | 19.68 (15.99-23.38) |
